# Overlaps in olfactive signalling coupled with geographic variation may result in localised pollinator sharing between closely related *Ficus* species

**DOI:** 10.1186/s12862-022-02055-0

**Published:** 2022-08-13

**Authors:** Xiaoxia Deng, Yufen Cheng, Yan-Qiong Peng, Hui Yu, Magali Proffit, Finn Kjellberg

**Affiliations:** 1 Guangdong Provincial Key Laboratory of Digital Botanical Garden and Key Laboratory of Plant Resource Conservation and Sustainable Utilization, South China Botanical Garden, The Chinese Academy of Sciences, Guangzhou, China; 2 CEFE, CNRS, Univ Montpellier, EPHE, Montpellier, IRD France; 3 CAS Key Laboratory of Tropical Forest Ecology, Xishuangbanna Tropical Botanical Garden, Chinese Academy of Sciences, Kunming, China

**Keywords:** Chemical signalling, Co-speciation, *Ficus hirta*, *Ficus triloba*, Mutualism, Speciation, *Valisia esquirolianae*, *Valisia javana*

## Abstract

**Background:**

In brood site pollination mutualisms, pollinators are attracted by odours emitted at anthesis. In *Ficus*, odours of receptive figs differ among species and the specific pollinators generally only enter figs of their host species ensuring a pre-zygotic barrier to plant interspecific hybridisation. However, field observations recorded that, in Guangdong province in China, *Valisia javana hilli*, the local pollinator of *F*. *hirta*, entered and reproduced successfully in the figs of the closely related *F. triloba* on a regular basis. We propose that closely related *Ficus* species produce similar receptive fig odours. Under particular contexts of odours locally present, the receptive fig odours of non-host figs of a *Ficus* species may become attractive to pollinators of closely related *Ficus* species. We used the headspace technique to collect in situ receptive fig odours of *F*. *triloba* in a series of locations in China. Under controlled conditions, we tested the attraction of fig pollinating wasps from *F. hirta* and *F. triloba* to host figs and non-host figs in Y tube experiments.

**Results:**

Receptive fig odours of *F. triloba* though different from those of *F. hirta*, were mainly composed of a same set of volatile organic compounds. When given the choice between receptive fig odours and air, the pollinating wasps were only attracted by their host’s odours. However, when given a choice between host and non-host figs the pollinators of *F. hirta* were equally attracted by the two odours while the pollinators of *F. triloba* tended to be more attracted by their host’s fig odours.

**Conclusions:**

Receptive fig odours vary geographically within species and the differentiation of receptive fig odours between closely related *Ficus* species is often incomplete. This allows localised or occasional pollinator sharing following different modalities. Cross stimulation when wasps are exposed simultaneously to odours of host and non-host species may be important. While occasional pollinator sharing may play a marginal role when wasp populations are robust, it may ensure the provisioning of new pollinators from the closest relative of a *Ficus* species if its pollinators go extinct.

## Background

Successful speciation involves establishing barriers to gene flow between incipient sister-species. While allopatric speciation is frequent, the distribution of sister species often strongly overlaps [1]. Therefore, reinforcement processes reducing genetic introgression may play a central role in speciation [2]. Sister species with overlapping ranges often occupy different ecological niches [1]. Models show that species coexist more easily if barrier reinforcement relies on habitat preferences rather than on species recognition [3]. In plants, the pre-zygotic barrier often involves change in pollinators [1], and pollinators may be habitat specialists [4].

Within this general context, systems in which plants associate with pollinators that breed in floral structures, *i.e.* brood pollination mutualisms, may ensure efficient pre-zygotic isolation among plant species. Indeed, the pollinators are often host specialists [5]. Plants typically attract their pollinating insect by releasing particular odours at anthesis that constitute species signatures [6]. Among such systems, figs and fig pollinating wasps provide an extreme case of specialised brood site pollination mutualism in which parallel cladogenesis between plants and insects has been the main form of diversification over the last 70 Ma [7]. They also provide a system where the range of plant species often strongly overlap. Indeed, sympatry is generalised among *Ficus* species [8]. Do brood site pollination mutualisms and *Ficus* in particular, follow the general rules associated with pre-zygotic barriers among related species, or do the particularities of these systems allow different diversification processes?

Fig pollinating wasps breed in the enclosed inflorescences (the figs) that characterise genus *Ficus*. The wasps are the sole pollinators of figs. Generally, a wasp species is associated with a single *Ficus* species, while a *Ficus* species is pollinated by a species or a species complex [9–12] and related *Ficus* species have related pollinator species [7]. The wasps are attracted to figs by a species-specific odour released when flowers are ready to be pollinated and receive wasp oviposition [13, 14]. Most *Ficus* species emit distinctive receptive fig odours [6], and wasps are sensitive to the ratio of different volatile organic compounds (VOCs) in the odour [13]. This allows high host-specificity.

The biology of the association suggests a simple, automatically enforced, reproductive isolation mechanism between incipient *Ficus* species. When the distribution of a *Ficus* species becomes fragmented (e.g. in glacial refugia during climatic oscillations), allopatric differentiation of pollinator and *Ficus* host may occur. If there is a local particularity in plant insect communication, i.e. in the odour emitted by receptive figs and in how it is interpreted by the wasps, this may result in a pre-zygotic barrier with respect to other populations surviving in other refugia [15]. In some *Ficus* species, receptive fig odours vary geographically [10, 16], pollinating wasp species vary geographically [9] and some wasps are attracted by the local odours of their host plant, and not by non-local odours [16]). If geographic receptive fig odour variation has a genetic basis, then a scenario of allopatric speciation in climatic refugia with geographic receptive fig odour differentiation instantly enforcing pre-zygotic isolation is plausible: in case of secondary contact between populations expanding from different refugia, the incipient species will remain distinct.

Receptive fig odours differ markedly between non-sister *Ficus* species and pollinators are not attracted by the odours of non-sister *Ficus* species (e.g*.*[17]). On the other hand, sister *Ficus* species may present similar odours, because of shared ancestry, and some pollinators are attracted by receptive figs of their host’s sister-species in experimental setups and/or in the field [18–20]. With receptive fig odours varying geographically within species and closely related species presenting similar receptive fig odours, we may expect a geographic patchwork of receptive fig odours, where receptive fig odours sometimes locally overlap sufficiently between closely related *Ficus* species to affect the specificity of wasp attraction.

*Ficus hirta* and *Ficus triloba* provide a model system to investigate such a situation. *Ficus hirta* presents clinal genetic variation across its range [9] and receptive fig odours diverge with increasing distance [16]. Throughout its range, it is pollinated by a set of parapatric wasp species of the *Valisia javana* species group. Its closest relative, *Ficus triloba*, occurs throughout most of that range and is pollinated by *Valisia esquirolianae* [21–23], a wasp that is closely related to the *Valisia javana* species groups [9] but is morphologically distinct [24]. In Guangdong province, South China, notably at Dinghu mountain, both *Valisia esquirolianae* and *Valisia javana hilli*, the local species of the *V. javana* complex [23] develop sucessfully in the figs of *Ficus triloba*. In samples collected throughout the range of *F. hirta*, *V. esquirolianae* was not found in the figs [9], though in the more recent survey it was obtained from some *F. hirta* figs in two locatities [23].

Here we test the hypothesis that (1) *F. hirta* and *F. triloba* receptive fig odours share some main compounds and that (2) in Dinghu mountain, receptive fig odours of *F. triloba* are attractive to *Valisia javana hilli,* while *V. esquirolianae* is not attracted by figs of *F. hirta*.

## Results

### Variation in scent profiles

The chemical composition of the odours emitted by receptive *F*. *triloba* figs is summarised per location in Table 1. Gas Chromatography-Mass Spectrometer (GC–MS) analysis revealed 46 compounds, with 20 compounds shared by all the locations. Based on their biosynthetic origin [25], the detected compounds fell into three distinct chemical classes: fatty acid derivatives, monoterpenes, and sesquiterpenes. The odours comprised 3 fatty acid derivatives, 8 monoterpenes, and 28 sesquiterpenes, and 7 compounds could not be identified. Ten compounds represented more than 5% of the odours in at least one location, namely α-cubebene, cyclosativene, α-copaene, β-cubebene, (E)-thujopsene, (E)- β-farnesene, (E)- β-caryophyllene, α-muurolene, germacrene D, δ-cadinene and unknown 6. All these compounds were also found at least once in *F. hirta* receptive fig odours [16].

**Table 1:** Composition of receptive *Ficus triloba* odours at the different sites

	RI	Shimen		DHS		XTBG	
		Occ	n = 13	Occ	n = 15	Occ	n = 4
*Fatty acid derivatives*							
1005	(E)-3-hexenyl acetate	9	0.77 ± 1.84	5	1.47 ± 3.92	4	1.26 ± 1.2
1102	Nonanal	6	0.38 ± 0.57	1	0 ± 0.01	0	
1203	Decanal	3	0.04 ± 0.1	1	0 ± 0.02	0	
	Sum		1.19		1.47		1.26
*Monoterpenes*							
934	α-Pinene	3	0.02 ± 0.05	1	0 ± 0.02	1	0.01 ± 0.01
973	Sabinene	2	0 ± 0.01	1	0 ± 0.02	0	
979	β-Pinene	0		1	0 ± 0	0	
984	6-Methyl-5-hepten-2-one	7	0.16 ± 0.2	7	0.17 ± 0.31	2	0.39 ± 0.74
991	β- Myrcene	3	0.01 ± 0.03	5	0.18 ± 0.31	0	
1030	Limonene	7	0.73 ± 1.2	6	0.18 ± 0.42	2	0.47 ± 0.84
1048	(Z)-β-Ocimene	2	0.03 ± 0.07	0		0	
1101	Linalool	4	0.03 ± 0.09	2	0.7 ± 2.61	0	
	Sum		0.98		1.23		0.87
*Sesquiterpenes*							
1343	δ-Elemene	10	0.92 ± 1.46	15	1.78 ± 1.61	4	1.25 ± 0.06
1355	α-Cubebene	10	1.16 ± 1.7	8	0.92 ± 2.4	4	5.4 ± 0.91
1365	α-Ylangene	9	1.11 ± 1.06	8	0.66 ± 1.3	1	0.01 ± 0.02
1375	Cyclosativene	11	1.49 ± 1.99	6	1.21 ± 3.08	4	0.37 ± 0.1
1382	Isoledene	9	0.38 ± 0.54	7	2.79 ± 8.58	0	
1384	α-Copaene	11	9.83 ± 14.8	15	7.11 ± 16.88	4	54.21 ± 7.5
1392	β-Bourbonene	11	1 ± 0.85	9	0.36 ± 0.49	4	0.05 ± 0.01
1387	β-Cubebene	8	0.92 ± 1.12	5	0.68 ± 1.52	4	2.63 ± 0.93
1389	β-Elemene	6	0.52 ± 0.8	11	1.5 ± 1.67	3	1.34 ± 2.63
1410	(E)-α- bergamotene	3	0.3 ± 0.71	1	0.1 ± 0.38	0	
1425	α-Cedrene	11	0.13 ± 0.13	6	0.18 ± 0.32	0	
1427	α-Gurjunene	4	0.1 ± 0.2	7	0.25 ± 0.91	3	0.03 ± 0.02
1429	cis-Thujopsene	10	18.32 ± 16.46	5	15.45 ± 25.21	0	
1430	(E)-β-caryophyllene	13	21.47 ± 8.68	11	27.16 ± 21.18	4	14.15 ± 4.05
1435	β-Copaene	11	1.21 ± 1.31	11	2.97 ± 2.27	4	2.21 ± 0.84
1440	(Z)- α-bergamotene	2	0.02 ± 0.04	2	0.01 ± 0.02	0	
1446	(Z)-β-farnesene	0		4	0.33 ± 0.7	0	
1454	Alloaromadendrene	5	0.3 ± 0.43	9	0.91 ± 0.82	3	0.51 ± 0.41
1457	(E)-β-farnesene	13	6.74 ± 3.65	13	4.87 ± 4.57	4	1.26 ± 1.35
1463	α-Humulene	8	2.73 ± 2.99	11	4.71 ± 3.62	3	1.48 ± 1.02
1482	γ-Muurolene	13	1.55 ± 0.56	12	3.79 ± 5.64	0	
1488	Germacrene D	11	6.55 ± 6.42	12	8.92 ± 8.18	4	5.33 ± 2.12
1494	α-Selinene	5	0.11 ± 0.19	1	0.28 ± 1.09	1	0.09 ± 0.18
1503	α-Bulnesene	6	0.15 ± 0.26	10	1.23 ± 1.83	0	
1505	α-Muurolene	13	5.16 ± 4.5	10	2.67 ± 2.64	4	1.99 ± 0.44
1510	β-bisabolene	4	0.72 ± 1.66	2	0.11 ± 0.39	0	
1520	γ-cadinene	10	0.54 ± 0.72	15	1.49 ± 2.14	4	0.03 ± 0.02
1528	δ-Cadinene	10	3.19 ± 3.82	11	1.99 ± 1.47	3	2.48 ± 1.78
	Sum		86.62		94.43		94.82
*Unknown compounds*							
1318	Unknown1	6	0.48 ± 0.98	2	0.04 ± 0.11	0	
1359	Unknown2	4	0.1 ± 0.29	0		0	
1378	Unknown3	5	0.36 ± 0.9	4	0.1 ± 0.19	0	
1395	Unknown4	10	0.83 ± 0.68	9	0.45 ± 0.87	1	0.01 ± 0.02
1451	Unknown5	10	1.59 ± 1.36	2	0.31 ± 1.17	0	
1465	Unknown6	13	7.11 ± 3.52	13	1.86 ± 1.49	4	3.04 ± 0.23
1480	Unknown7	10	0.73 ± 1.13	7	0.11 ± 0.2	2	0.01 ± 0.01
	Sum		11.2		2.87		3.06
*Main fragments (m/z) for unknown compounds*							
Unknown1		121, 93, 90, 91, 80, 105, 75, 133, 60, 107					
Unknown2		189, 147, 40, 133, 38, 133					
Unknown3		161, 105, 55, 121, 45, 91					
Unknown4		119, 93, 90, 91					
Unknown5		162, 147, 70, 105, 50, 91/					
Unknown6		93, 91, 60, 105, 40, 79					
Unknown7		69, 93, 95, 105, 85, 91					

Relative proportions of the volatile organic compounds are indicated (mean ± SD per site). RI = Retention Index; Occ = occurrence of each VOC, total and by population; n = number of samples

**Fig. 1:**
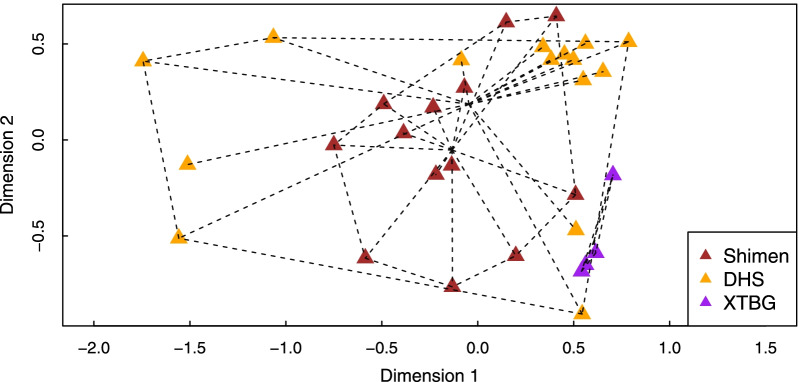
NMDS representation of the relative proportions of VOCs in the odors emitted by *Ficus triloba. *The Bray-Curtis dissimilarity Index was used. Each triangle represents an individual. Colour indicates sampling location ( Shimen, Shimen National Forest Park—brown; DHS, Dinghu mountain—orange; XTBG, Xishuangbanna Tropical Botanical Garden—purple.) Odour profiles vary significantly between each of the three study sites (PERMANOVA: F_(2;31)_ = 3.6554, P = 0.001) (stress = 0.124)

The non-metric multidimensional scaling (NMDS) (stress = 0.124) applied to the *Ficus triloba* dataset (i.e., the relative proportion of each VOC in the odour emitted by each studied sample) showed that, while many point overlapped among locations, the odours of receptive figs differed significantly among locations (Fig. 1; Permutational multivariate analysis of variance (PERMANOVA): F_(2;31)_ = 3.6554, P = 0.001). Pairwise comparisons using permutation MANOVAs on the pairwise distance matrix between localities showed differences between all the localities (P < 0.05). The dispersion of the VOC profiles was significantly heterogeneous among the three locations sampled (F_2,29_ = 4.3772, P = 0.03), but not between Shimen and DHS (F_1,26_ = 0.7443, P = 0.3962). The results within location at Shimen and even more at DHS presented a large variance.

### Inter-specific variation in the chemical message emitted by receptive figs

**Fig. 2:**
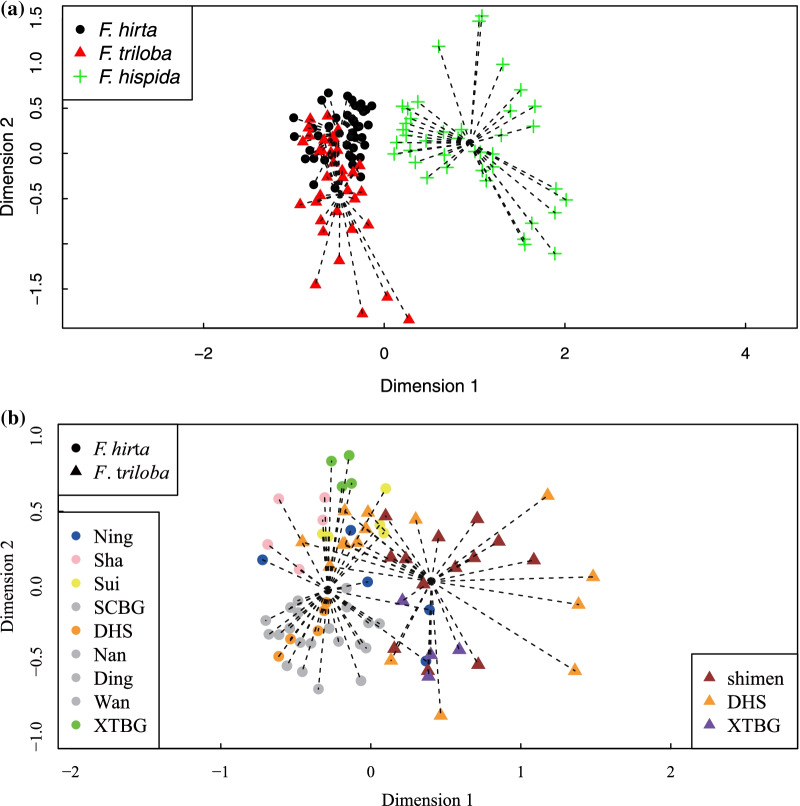
Comparison of receptive fig odours among species. 2a: comparison between *Ficus triloba*, *F. hirta* and *F. hispida*. Black, red and green dots represented *F. hirta*, *F. triloba*, *F. hispida*, respectively. 2b comparison between *F. triloba* and *F. hirta*. Non-metric multi-dimentional scaling (NMDS) representation of the relative proportions of VOCs in the odors emitted by individual fig plants showing groupings according to location based on Bray–Curtis dissimilarity Index (stress = 0.18 for 2a and stress = 0.204 for 2b)

**Fig. 3:**
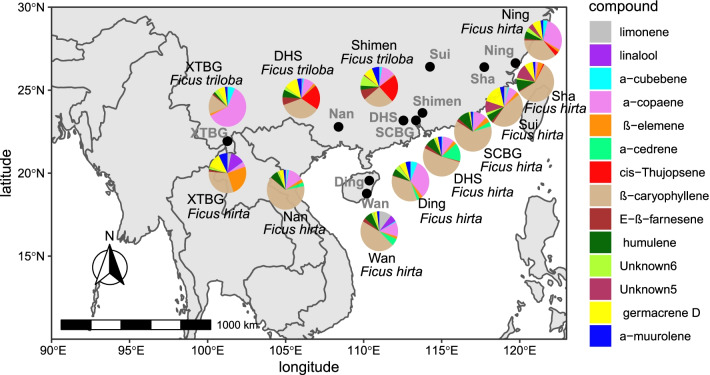
Geographic variation in receptive fig odour composition for *Ficus hirta* and *Ficus triloba*. The solid black circle represents the location of each population. Colour within the pie shows volatile organic compounds representing more than 5% of the odor in at least one location in at least one species. The color within the pie shows the proportional contribution of different volatile compounds. Odors of *F. hirta* and *F.triloba* are marked with black and blue letters in the pie chart at the location

**Fig. 4:**
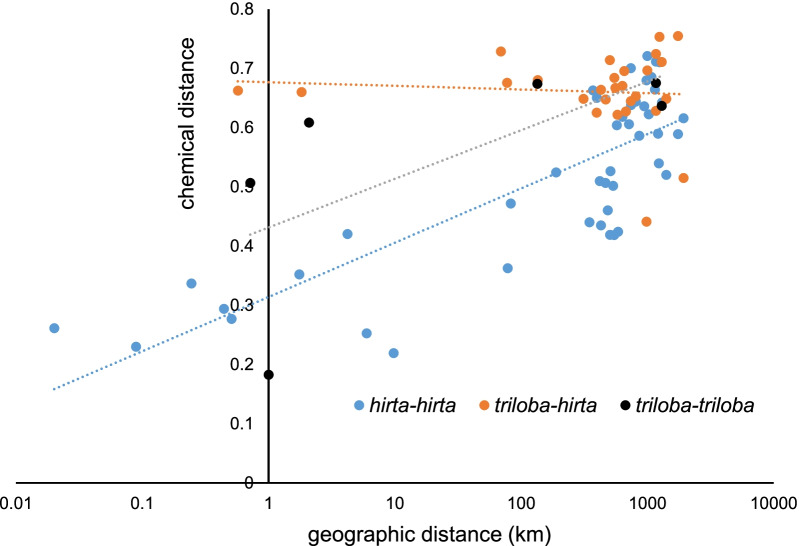
Correlation between geographic distance and chemical distance, within and between species. The regression lines and coefficient of determination are for log(distance)

All the compounds representing more than 5% of the odour of *F. triloba* in at least one location were also detected in the odours produced by at least one individual of *F. hirta* (Table 2). Reciprocally all the compounds representing more than 5% of the odour of *F. hirta* in at least one location were also detected in at least one individual of *F. triloba* (Table 2). In contrast, out of these 17 compounds, 12 were not detected in *F. hispida* odours, while 5 compounds representing each over 5% of receptive fig odours of *F. hispida* in at least one location where not detected in *F. triloba* and *F. hirta* odours. In agreement, in the NMDS plot including the 3 species, there was a large overlap between *F. triloba* and *F. hirta* odours, while *F. hispida* was separated (Fig. 2a). Nevertheless, receptive fig odour differed between *F. hirta* and *F. triloba* (PERMANOVA, F _(1,79)_ = 9.65, P = 0.001, Fig. 2b) despite 28 shared compounds (Tables 1, 2; [16]). Geographic variation in receptive fig odours for *F. hirta* and *F. triloba* are presented in Fig. 3. The chemical distance between *F. hirta* samples from different locations increases with log distance (linear regression between chemical distance and log geographic distance, R^2^ = 0.65 p < 10^–10^, Fig. 4) while the chemical distance between *F. triloba* and *F. hirta* odours was independent of geographic distance between sampling locations (linear regression between chemical distance and log geographic distance, R^2^ = 0.006, P = 0.70, Fig. 4). The chemical distances beween *F. hirt*a and *F. triloba* odours within location at XTBG and at DHS (the two values for low geographic distance in Fig. 4) were close to the median value of interspecific comparisons, with 14 higher values in comparisons between *F. triloba* and *F. hirta* odours from different locations and 11 lower values. More locations need to be sampled to test for a correlation between geographic distance and chemical distance of receptive fig odours of *F. triloba*.

**Table 2:** Receptive fig odour at the different sites for the different species

Species	Locality	Limonene	Linalool	α-cubebene	cyclosativene	α-copaene	β-cubebene	β-elemene	α-cedrene	cis-Thujopsene	β-caryophyllene	€-β-farnesene
*F.hirta*	Ning	0.1	0.0	3.1	1.7	23.3	1.5	1.7	0.1	2.9	29.1	1.2
*F.hirta*	Sha	0.1	0.0	0.0	0.2	1.7	0.0	3.5	0.0	0.8	44.6	0.6
*F.hirta*	Sui	2.5	0.0	0.8	1.7	5.6	0.4	2.2	0.0	0.0	34.0	1.3
*F.hirta*	SCBG	0.0	0.0	0.6	1.4	10.4	0.0	4.0	3.3	0.0	56.0	2.2
*F.hirta*	DHS	0.0	0.0	0.8	0.4	8.9	0.0	2.7	13.3	0.5	46.4	2.7
*F.hirta*	Nan	1.4	0.1	1.1	2.0	12.0	0.4	2.5	3.0	0.0	57.3	0.2
*F.hirta*	Ding	0.4	0.0	4.8	0.4	27.5	1.0	2.5	2.1	0.0	29.8	0.4
*F.hirta*	Wan	9.5	5.2	0.7	1.2	10.6	0.1	1.9	5.2	0.0	40.7	2.7
*F.hirta*	XTBG	2.4	8.9	0.2	0.8	3.0	0.0	18.3	0.0	0.0	22.9	1.2
*F.triloba*	shimen	1.3	0.0	0.7	1.3	6.3	0.7	0.5	0.2	21.0	19.7	6.6
*F.triloba*	DHS	0.3	0.0	0.1	3.2	2.2	0.3	2.2	0.2	0.0	40.3	2.8
*F.triloba*	XTBG	0.5	0.0	5.4	0.4	54.2	2.6	1.3	0.0	0.0	14.2	1.3
*F.hispida*	SCBG	0.9	**0.0**	**0.0**	**0.0**	**0.0**	**0.0**	8.4	**0.0**	**0.0**	14.8	3.2
*F.hispida*	Nan	6.3	**0.0**	**0.0**	**0.0**	**0.0**	**0.0**	22.2	**0.0**	**0.0**	31.5	2.4
*F.hispida*	Ding	0.0	**0.0**	**0.0**	**0.0**	**0.0**	**0.0**	3.9	**0.0**	**0.0**	31.3	3.8
*F.hispida*	XTBG	1.0	**0.0**	**0.0**	**0.0**	**0.0**	**0.0**	38.6	**0.0**	**0.0**	8.0	15.3
*F.hispida*	Thailand	0.4	**0.0**	**0.0**	**0.0**	**0.0**	**0.0**	4.7	**0.0**	**0.0**	4.2	9.8

Species	Locality	humulene	Unknown6	Unknown5	germacrene D	α-muurolene	δ-cadinene	(Z)-3-Hexenyl acetate	β-Myrcene	(E)-β-Ocimene	α-Copaene	Unknown c
*F.hirta*	Ning	4.9	2.7	3.6	5.9	1.7	2.6	**0.0**	**0.0**	**0.0**	**0.0**	**0.0**
*F.hirta*	Sha	7.2	0.9	9.2	5.8	1.6	1.1	**0.0**	**0.0**	**0.0**	**0.0**	**0.0**
*F.hirta*	Sui	4.6	1.7	7.2	12.4	2.8	1.4	**0.0**	**0.0**	**0.0**	**0.0**	**0.0**
*F.hirta*	SCBG	8.9	0.5	0.0	1.0	1.7	1.0	**0.0**	**0.0**	**0.0**	**0.0**	**0.0**
*F.hirta*	DHS	7.1	1.1	0.1	2.2	2.8	1.3	**0.0**	**0.0**	**0.0**	**0.0**	**0.0**
*F.hirta*	Nan	6.2	0.9	0.3	3.8	1.6	1.2	**0.0**	**0.0**	**0.0**	**0.0**	**0.0**
*F.hirta*	Ding	4.8	3.3	0.2	5.0	3.6	4.1	**0.0**	**0.0**	**0.0**	**0.0**	**0.0**
*F.hirta*	Wan	6.1	1.8	0.3	2.2	1.8	2.6	**0.0**	**0.0**	**0.0**	**0.0**	**0.0**
*F.hirta*	XTBG	0.3	1.2	0.0	9.1	5.5	1.5	**0.0**	**0.0**	**0.0**	**0.0**	**0.0**
*F.triloba*	shimen	2.4	8.2	0.6	7.0	5.6	4.9	**0.0**	**0.0**	**0.0**	**0.0**	**0.0**
*F.triloba*	DHS	7.2	2.1	0.2	16.8	3.5	2.7	**0.0**	**0.0**	**0.0**	**0.0**	**0.0**
*F.triloba*	XTBG	1.5	3.0	0.0	5.3	2.0	2.5	**0.0**	**0.0**	**0.0**	**0.0**	**0.0**
*F.hispida*	SCBG	4.6	**0.0**	**0.0**	**0.0**	**0.0**	**0.0**	20.3	4.9	4.1	7.1	4.3
*F.hispida*	Nan	6.2	**0.0**	**0.0**	**0.0**	**0.0**	**0.0**	0.0	0.0	7.0	9.6	0.4
*F.hispida*	Ding	6.4	**0.0**	**0.0**	**0.0**	**0.0**	**0.0**	0.7	5.7	5.4	14.1	0.1
*F.hispida*	XTBG	2.4	**0.0**	**0.0**	**0.0**	**0.0**	**0.0**	7.8	2.0	1.6	2.8	5.4
*F.hispida*	Thailand	1.0	**0.0**	**0.0**	**0.0**	**0.0**	**0.0**	20.5	7.0	31.7	2.2	0.1

The numbers correspond to the mean relative proportion of each Volatile Organic Compounds representing more than 5% of the odours in a least one location in at least one species. Data for *Ficus hirta* from (16). Values in bold indicate compounds that were not observed in any sample of a species. Note the strong difference between *F. triloba-hirta* and *F. hispida*

### Insect behavioral tests

**Fig. 5:**
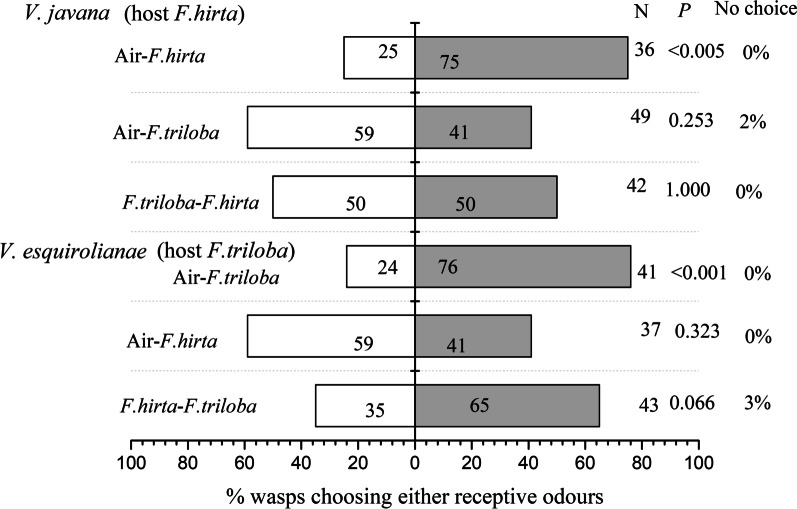
Wasp choices when confronted with different odours in a Y-tube olfactometer. We used binomial tests for statistical comparisons between the number of choices for receptive fig odour versus clean air or choice between receptive fig odours of the two *Ficus* species. N: number of tested wasps. P: probability, two tailed

Results of Y-tube olfactometer tests are presented in Fig. 5. When given the choice between the odour of receptive figs against cleaned air, both *V. javana hilli* and *V. esquirolianae* were attracted by the receptive fig odour of their host species (two-tailed binomial test, P < 0.005, n = 36 and P < 0.001, n = 41, respectively) and they were not attracted by the receptive fig odours of the other species (two-tailed binomial test, P = 0.253; n = 49, and P = 0.323, n = 37, respectively).

However, when *Valisia javana hilli* were first exposed to a mix of receptive fig odours of the two *Ficus* species in the first section of the Y tube olfactometer, they became equally attracted by the two branches of the olfactomer although one presented a flow of *F. hirta* odours and the other one of *F. triloba* odours (two tailed binomial test, P = 1; n = 42). Exposed to the same conditions, *Valisia esquirolianae* tended to be more attracted by the odours of *F. triloba* (two-tailed binomial test, P = 0.066; n = 43).

## Discussion

Despite significant differences in receptive fig odours between *F. hirta* and *F. triloba*, there was a large overlap in the VOCs constituting these odours. All the compounds present at a concentration above 5% in at least one location in one species were also detected in the other species. This overlap was much more marked than with the VOCs constituting the receptive fig odours of *F. hispida* suggesting an effect of phylogenetic distance. A similar situation was observed for the species group of *Ficus itoana* (subgenus *Sycomorus*) in Papua New-Guinea, with overlap of receptive figs odours of the species group on an NMDS plot and separation from other species of subgenus *Sycomorus* belonging to other sections [20].

The similarity in the main VOCs constituting the odours suggests that the pollinating wasps of both *F. hirta* and *F. triloba* are capable of detecting some of the VOCs composing the receptive fig odour of the other *Ficus* species. If, by chance, the relative proportions of the VOCs the wasps detect, are locally sufficiently similar between the two species, then the wasp may be attracted to the usually non-host species [13]. On the other hand, the wasps, with their limited repertoire of olfactory genes [26], may not have the olfactory receptors allowing them to detect the VOCs constituting receptive fig odours of *F. hispida*.

The difference between receptive fig odours in *F. hirta* increased with geographic distance [16] while the difference between *F. hirta* and *F. triloba* receptive fig odours was independent of geographic distance. This suggests lack of interference between the two species in the local evolution of their receptive fig odours. We suggest that independent odour variation, of genotypic or phenotypic origin, in the two species may lead to occasional situations of local overlap of the part of the chemical message detected by one or the other species of wasp.

In the Y tube experiment, when wasps were given the choice between purified-air and receptive fig odours, they were attracted by their host species’ figs and were not attracted by non-host figs.

However, when the wasps were first exposed to a mix of odours of the two species, *Valisia javana hilli* became attracted by figs of both species. We propose that during the exposition to the mix of odours, *F. hirta* odours stimulate *Valisia javana hilli* so that it subsequently responds to the previously non-attractive odour of *F. triloba*. Such situations could occur under natural conditions, as we have several times observed *F. hirta* growing under the cover of *F. triloba*. In a similar process, male European corn borer moths are initially highly discriminative according to VOC relative concentrations in pheromones. However, after initial stimulation, they respond to a broader range of relative concentrations [27].

The attraction of host specialist pollinating wasps by receptive figs of closely related *Ficus* species has previously been investigated in Y tube olfactometer experiments for three situations. *Ficus boninsimae* and *F. nishimurae* are two very closely related species co-occurring in the Ogasawara islands, Japan. *Ficus boninsimae* is an open habitat species while *F. nishimurae* is an understory tree. In Y tube experiments, pollinators of *F. boninsimae* were equally attracted by figs of *F. boninsimae* and *F. nishimurae*, while the pollinators of *F. nishimurae* were more attracted by *F. nishimurae* fig odours [18]. In Papua New Guinea, the closely related *F. microdyctia*, *F. sp.* and *F. itoana* replace each other along an altitudinal gradient. Their receptive fig odours overlap in an NMDS plot. In Y tube tests against air, the pollinator of *Ficus sp.* was attracted by fig odours of *F. sp* and of *F. microdyctia*, but not those of *F. itoana*. The pollinator of *F. itonana* was attracted by receptive fig odours of *F. itoana*, but not those of the two other species. Finally, the pollinator of *F. microdyctia* was avoiding the odours of receptive figs of *F. sp.* and *F. itoana* [20]. *Ficus semicordata semicordata* and *F. s. montana* co-occur from Nepal to Laos through South-China but have distinct habitats [19]. Receptive fig odours of *Ficus s. semicordata* are mainly constituted by a highly unusual compound, p-methylanisole [14], and this compound was also found in receptive fig odours of *F. s. montana* individuals. Pollinators of *F. s. semicordata* were preferentially attracted by their host species when given a choice, but when given no choice, they were attracted by non-host figs. Pollinators of *F. s. montana* were equally attracted by receptive figs of the two varieties. Finally, the ranges of *Ficus auriculata*, *F. oligodon* and *F. hainanensis*, which form a species complex, overlap throughout continental Asia but they occupy distinct habitats [28]. They share pollinators throughout their regions of co-occurrence and the receptive fig odours of *F. auriculata* and *F. oligodon* were not distinguishable [28].

Hence, although all investigated sister *Ficus* species that occur in sympatry present similarities in their fig odours, they occupy different habitats. Generally, they do not share pollinators, but their pollinators may be attracted by non-host receptive figs in Y tube experiments, following variable modalities and directionality. There is no evidence supporting selection for divergence in olfactive signalling between these closely related *Ficus* species and there is no evidence supporting selection on the wasps to use several hosts. All the investigated cases involve dioecious *Ficus* species, in which pollinator dispersal is limited [29]. Hence, for dioecious *Ficus* species, habitat differentiation between closely related species may constitute the main barrier to gene flow between species. Pollinator specificity is a complementary force, but it is leaky. As such, *Ficus* follow the general pattern of separation between closely related species in the tree of life [1].

On islands, small population sizes may lead to local extinctions of pollinators. In such situations, because of the limited barriers to wasps detecting receptive figs of close relatives of their usual host species, recolonization of a *Ficus* species by pollinators of a close relative is expected. This is the case in Taiwan where *Blastophaga nipponica* pollinates *Ficus erecta* as elsewhere, but distinct host-races of *B. nipponica* pollinate the more localised *F. formosana* and *F. tannoensis* [30]. In an artificial situation in Hawaii, *Ficus rubiginosa* was introduced with its pollinator *Pleistodontes imperialis*. *Ficus watkinsiana*, a close relative of *F. rubiginosa* was also introduced. It is now beginning to be pollinated by *P. imperialis*, while in their native range the two *Ficus* species co-occur and are pollinated by different wasp species [31]. Hence, the barrier to colonisation of closely related host species by a same wasp species could often be competition with the established populations of pollinating wasp. Reciprocally, when a *Ficus* species is introduced into a part of the world where no closely related species sustains a population of wasps, it will remain unpollinated as long as its pollinator is not introduced [32]. Within this perspective, specialised pollination in *Ficus* may limit their invasiveness when introduced into new parts of the world as long as pollinators from their continent of origin are not introduced.

## Material and methods

### Study system and collection sites

*Ficus triloba* Buch.-Ham. ex Voigt (= *Ficus esquiroliana* Léveillé) (subg. *Ficus*, sect. *Eriosycea*, subsect. *Eriosycea*) is a dioecious tree up to 15 m tall while *Ficus hirta* is a small shrub [32–34]. *Ficus triloba* male trees produce a single main crop releasing pollen loaded wasps in July in time to pollinate the main crop on female trees that ripens in September–October [33]. *Ficus hirta*, its closest relative [21, 22]*,* produces figs year-round, with seasonal peaks, in June-July, and in October–November [36, 37] thus overlapping with *F. triloba* phenology. *Ficus triloba* has large figs, about 30 mm in diameter at receptivity [34], while those of *F. hirta* are about 10-15 mm [38]. Receptive figs of *F. triloba* emit a strong floral scent while the smell of receptive figs of *F. hirta* is hardly detectable by the human nose [22]. *Ficus hirta* is pollinated by a set of 9 different wasps throughout its distribution [9], while a same pollinator (*Valisia esquirolianae*) has been collected on *F. triloba* in Taiwan, in continental China, and in Thailand [24, 39]. The two species are sympatric across most of their distributions that extends from northeast India and subtropical China to the Malay Peninsula [35]. While their habitats differ, the two species may grow side by side in secondary habitats, for instance in abandoned tree plantations or close to each other as in our study sites in Dinghu Mountain (DHS, a National Nature Reserve, established in 1956) and in Shimen (a forest park established in1995) in Guangdong province, China. In these two sites, *V. javana hilli* was observed to develop in figs of *F. triloba* [23].

Between November 2017 and June 2019, in wet (May to September) and dry (November to March) season, to explore the diversity of receptive fig odours, we collected receptive fig odours from *Ficus triloba* at DHS, Shimen and at the Xishuangbanna Tropical Botanical Garden (XTBG) in Yunnan. We collected 15 samples in the region of Dinghu mountain (DHS, 112.54 E, 23.16 N), 13 samples in Shimen National Forest Park (Shimen, 113.45 E, 23.27 N), and 4 samples in the Xishuangbanna Tropical Botanical Garden (XTBG,101.15 E, 21.55 N).

### Volatile collections

The chromatoprobe head-space method, which was initially used in *Silene*, was adopted to collect fig odours in situ [16, 40, 41]. Odour collection was performed outdoors in the shade between 10:00 a.m. and 5:00 p.m. on sunny days, corresponding to the insects’ period of peak activity during our field season. Five-15 receptive figs were enclosed together in a polyethylene terephtalate (Nalophan®, Kalle Nalo GmbH, Wursthüllen, Germany) bag for 30 min. Then, air was pulled out of the bag for 5 min (flow rate: 200 mL/min) through a filter filled with 1.5 mg of Carbotrap 20–40 and 1.5 mg of Tenax 60–80, in which the volatile organic compounds (VOCs) were trapped. In parallel, at every collection, we made a ‘blank’ extraction from a bag that contained no fig, using the same protocol. One microliter of a solution of internal standards (n-Nonane and n-Dodecane, 110 ng/μl of each) was added to each filter before scent extraction, so that we could control for VOC loss during storage and transport. The samples were stored at − 20 °C until VOC analysis.

### VOC analysis

The samples were analysed using gas chromatography coupled with mass spectrometry and the compounds were identified as detailed in Deng et al. 2021. We obtained a global dataset, where the composition of the odour extracted from each sample is expressed by the relative proportions of each VOC in the emitted odour (semi-quantitative data). This dataset was complemented by previous data obtained from *Ficus hirta* [16] to compare the odours of the two species, and from *F. hispida* (subgenus *Sycomorus*) (Deng et al. submitted) another sympatric species to provide an outgroup.

Divergence in chemical profiles across locations was estimated with non-metric multidimensional scaling (NMDS) in two dimensions, based on a Bray–Curtis similarity matrix, using the R-package vegan [42]. Pairwise distance between individuals for relative proportions of VOCs was calculated using the Bray–Curtis dissimilarity index, which ranges between 0 and 1. Chemical distance matrices were calculated with the function “vegdist” between locations and between species, using available data for *F. hirta* [16], Two-dimensional plots were constructed using the “metaMDS” function algorithm after data standardization with “decostand” function in R (v. 3.5.1). A stress value is given, indicating how well the particular configuration represents the distance matrix (stress values < 0.2 are desirable). To test if the variation in chemical composition between locations was significant, we carried out permutational multivariate analysis of variance tests (PERMANOVA) on the distance matrix using the function “adonis” in the vegan package [42]. The model used 999 permutations, and we FDR corrected p-values to control for multiple comparisons. Multivariate homogeneity of group dispersions (variances) was tested using the “betadisper” function. SIMPER (similarity percentage) was used to identify the compounds responsible for dissimilarities between groups.

### Insect behavioural tests

In Dinghu Mountain (DHS), *Valisia javana hilli* was observed to develop in figs of *F. triloba* along with *V. esquirolianane*. On the contrary, *V. esquirolianae* was not observed to develop in the figs of *F. hirta* at DHS [23].

In order to test if the local populations of *Valisia esquirolianae* and *V. javana hilli* are attracted by the odours released by receptive figs of *F. triloba* and *F. hirta*, wasp attraction was tested using Y-tube olfactometers in DHS. Bioassays were conducted outside, on a sunny day between 9 and 12 a.m. We tested the response of the wasps when given the choice between floral odours emitted either by *F. triloba* or by *F. hirta* and filtered air (i.e. control), and their response to a choice between the floral odours of the two species. Three different series of tests were used: receptive figs of host versus control, receptive figs of non-host versus control and receptive figs of host versus receptive figs of non-host. We used the same size Y-tube olfactometer (stem 8 cm; arms 9 cm; diameter 1.5 cm) as Proffit et al. (2009) to test the attraction of the pollinating wasps of *F. hispida*. Humidified air was purified with activated charcoal and blown into a glass vial connected to each lateral arm (200 ml/min per arm). The vial connected to one arm contained receptive figs stemming from several trees, and in the other, the vial was either empty (in controls) or it contained receptive figs of the other species. For tests involving *Ficus triloba*, 2 receptive figs were put into the vial, while for *F. hirta*, 4 receptive figs were put into the vial. When comparing the attraction by receptive figs of the two species, due to the large difference in size, an equal weight of fresh figs was used. To ensure continuous odour production, we changed the odour source every two hours. Wasps were introduced individually into the central arm of the Y-tube and their movements were recorded for 10 min. To avoid a potential directional bias, the directions of control and odour source were reversed after each trial. To eliminate scent contamination, the Y-tubes were cleaned with pure acetone before each trial, as was the entire network of connecting tubes after each five trials. The observer noted the behavioural choice made by each individually tested fig wasp for 10 min among three modalities: choice for odour, choice for control, or no choice. We considered that wasps made no choice when they stayed motionless for 3 min in the departure section and/or the central arm before the bifurcation of the olfactometer. All the adult female fig wasps were newly emerged from male figs. For each experiment, we used two-tailed binomial tests (with a probability of 0.5) to compare the number of choices for odour versus choices for no odour or other odour (excluding the no-choice response).

## Data Availability

All figs sample collection permissions or licenses were obtained. All datasets generated or analyzed during this study are included in this published article.

## Abbreviations

GC–MS: Gas Chromatography-Mass Spectrometer
NMDS: Non-metric multidimensional scaling
VOCs: Volatile organic compounds
PERMANOVA: Permutational multivariate analysis of variance
